# Recent Advances in Silicon Nanomaterial-Based Fluorescent Sensors

**DOI:** 10.3390/s17020268

**Published:** 2017-02-03

**Authors:** Houyu Wang, Yao He

**Affiliations:** Jiangsu Key Laboratory for Carbon-Based Functional Materials and Devices, Institute of Functional Nano and Soft Materials (FUNSOM), Soochow University, Suzhou 215123, China; hywang@suda.edu.cn

**Keywords:** silicon nanomaterials, fluorescence, sensors

## Abstract

During the past decades, owing to silicon nanomaterials’ unique optical properties, benign biocompatibility, and abundant surface chemistry, different dimensional silicon nanostructures have been widely employed for rationally designing and fabricating high-performance fluorescent sensors for the detection of various chemical and biological species. Among of these, zero-dimensional silicon nanoparticles (SiNPs) and one-dimensional silicon nanowires (SiNWs) are of particular interest. Herein, we focus on reviewing recent advances in silicon nanomaterials-based fluorescent sensors from a broad perspective and discuss possible future directions. Firstly, we introduce the latest achievement of zero-dimensional SiNP-based fluorescent sensors. Next, we present recent advances of one-dimensional SiNW-based fluorescent sensors. Finally, we discuss the major challenges and prospects for the development of silicon-based fluorescent sensors.

## 1. Introduction

Rapid, sensitive, and accurate detection of chemical and biological species is of essential importance for various fields, such as biomedical diagnosis, drug screening, food safety, environmental protection, homeland and international security, among others [1,2,3,4,5]. Even though a number of sensors (e.g., bioassay kits) are commercially available, novel kinds of sensors with ease of portability, sufficient sensitivity, high specificity, excellent reproducibility, multiplexing detection capability, and low cost remain in high demand.

Optical sensors and electrochemical sensors are commonly used sensors. The optical sensors can be divided into different types based on different detection signals, such as fluorescence, Raman, surface plasmon resonance (SPR), and so forth. Among these, fluorescent sensors with high sensitivity, short response time, easy portability, minimal equipment requirements, and low cost have attracted extensive attention [6,7,8,9,10]. Recently, with the significant development of optical nanomaterials, fluorescence-sensing has greatly benefited from various kinds of zero-dimensional luminescent nanoparticles. Among them, fluorescent colloidal II–VI quantum dots (QDs) have drawn much attention due to their unique optical merits, such as high photoluminescence quantum yield (PLQY), robust photostability, and sharp and tunable emission spectra, thereby allowing prolonged and multiplexed detection [11,12,13]. While these QD-based nanosensors are suitable for sensing various species, people’s concern regarding the heavy metal content of II–VI QDs limit their wide-scale use, since the heavy-metal content might produce inevitable toxicity to human health and lead to environmental contamination [5,14]. As a result, the demand for developing novel kinds of fluorescent sensors made of heavy-metal-free fluorescent nanomaterials with good biocompatibility has been on the rise.

On the other aspect, silicon, as the second-most abundant element in earth’s crust (28% by mass, with oxygen being the most abundant) guarantees a rich and low-cost resource support for a variety of silicon-related applications, especially for the modern semiconductor industry [15]. Impressively, distinguished from the poor photoluminescence of bulk silicon due to its indirect bandgap, nanometric-sized silicon shows distinct photoluminescence that benefits from the quantum confinement effect when the particle size is generally less than 5 nm [16,17]. In addition to the strong fluorescence, the silicon nanomaterials usually feature photostability superior to that of common organic dyes and most semiconductor nanomaterials. For instance, upon high-power UV irradiation, in comparison to rapid fluorescence quenching of fluorescein isothiocyanate (FITC) (i.e., the fluorescence quickly quenches after 5 min of UV irradiation) and relatively stable CdTe QDs or CdSe/ZnS QDs (i.e., the fluorescence can be retained for 120 min under UV irradiation), the prepared fluorescent silicon nanoparticles can preserve strong and stable fluorescence under high-power UV irradiation for 400 min [14]. More significantly, silicon nanomaterials (e.g., silicon nanoparticles (SiNPs), silicon nanowires (SiNWs), silicon nanorods (SiNRs), etc.) have little or no toxicity due to the benign biocompatibility of silicon, which is highly attractive for biomedical and biological applications. Recent studies have demonstrated that silicon nanomaterials (e.g., SiNPs, silicon nanoneedles) were easily biodegradable and decomposed into silicic acid, which can be subsequently cleared from the body via kidney without apparent toxicity [18,19]. The unique optical properties combined with benign biocompatibility offer exciting opportunities for design of high-performance fluorescent sensors [20,21,22].

**Figure 1:**
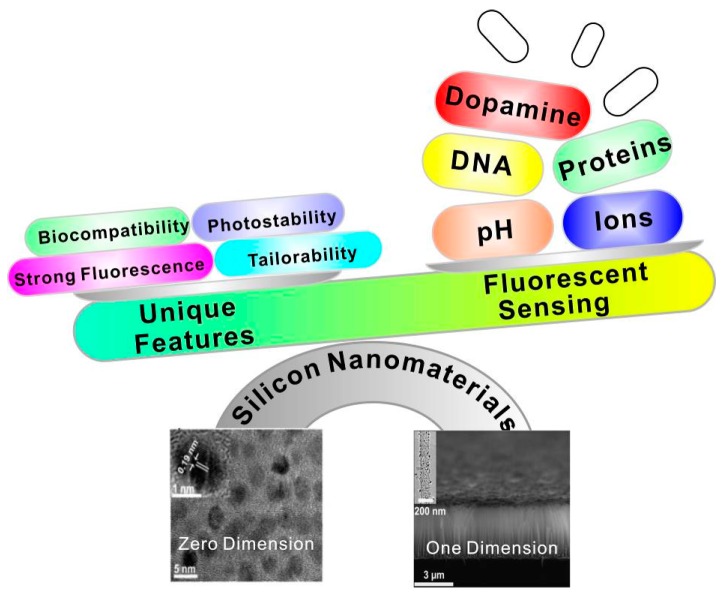
Silicon nanomaterial-based fluorescent sensors constructed by using zero- and one-dimensional silicon-based nanomaterials featuring several merits, and their use for various sensing applications. (Zero-dimensional silicon nanoparticles (SiNPs) image was reproduced with permission from ref. [23]. Copyright 2015, American Chemical Society. One-dimensional silicon nanowires (SiNWs) image was reproduced with permission from [24]. Copyright 2014, John Wiley & Sons, Inc.)

Consequently, during the last decade, we have witnessed vast advancements in the development of silicon nanomaterial-based optical analytical platforms for sensing various chemical and biological species, including chemical reagents, metal ions, proteins, intracellular pH, and so forth. To date, there are some review-type articles mainly focusing on the introduction of zero-dimensional silicon quantum dot-based sensors, omitting the significant works of one-dimensional silicon nanomaterial-based fluorescent sensors [20,21,22]. Hence, in this review, we will introduce the latest representative achievements of zero/one-dimensional silicon nanomaterial-based fluorescent sensors in detail. As summarized in Figure 1, according to different dimensions of nanostructures, the silicon nanomaterial-based fluorescent sensors mentioned in this review cover zero- and one-dimensional fluorescent silicon nanosensors. We further discuss the major challenges and prospects for the development of silicon nanomaterial-based sensors in the final section.

## 2. Preparation of Silicon Nanomaterials

To date, numerous synthetic strategies have been proposed for the preparation of these silicon nanostructures. Since the topic of this review is not synthetic methods of silicon nanomaterials, we just briefly introduce recent representative strategies for preparation and functionalization of silicon-based nanomaterials. Readers who are interested in detailed fabrication protocols can refer to several excellent review published recently [14,20,25,26].

### 2.1. Preparation of Zero-Dimensional Silicon Nanomaterials

Fluorescence zero-dimensional SiNPs featuring unique optical properties (i.e., bright luminescence and a strong antiphotobleaching property) and feeble toxicity have extensively been exploited for fabricating new fluorescent nanosensors. So far, several synthetic approaches have been well developed for preparation of SiNPs, including laser pyrolysis [27], electrochemical and chemical etching [28,29], microemulsion [30,31], high-temperature processes [32], plasma-assisted synthesis [33,34], and so forth. Afterwards, in order to meet requirements of different applications, the as-prepared SiNPs need further surface modification, such as surface oxidation and etching [35], hydrosilylation [36], bioconjugation [23,37,38], and so forth.

**Figure 2:**
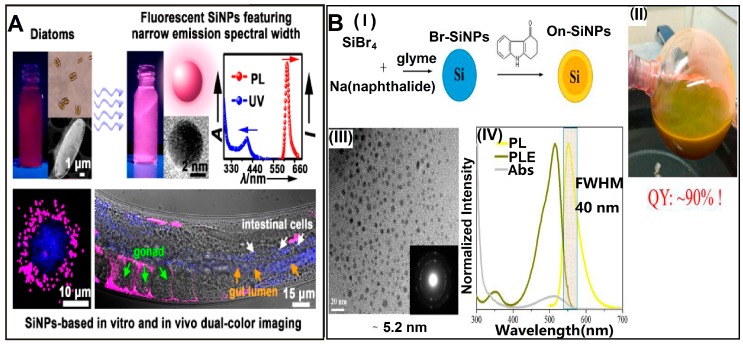
(**A**) Schematic illustration of biomimetic synthesis of fluorescent SiNPs and their use for in vitro and in vivo dual-color imaging. Reproduced with permission from ref. [38]. Copyright 2015, American Chemical Society; (**B**) schematic illustration of synthesis of yellow-emitting SiNPs with ultrahigh quantum yield and narrow photoluminescence (PL) bandwidth; (**I**) scheme of synthetic and modified procedures; (**II**) photograph of SiNPs in glyme under ambient light; (**III**) TEM image of SiNPs. Inset is the selected-area electron diffraction pattern of SiNPs; IV) PL, photoluminescence excitation (PLE), and absorbance (Abs) spectra of SiNPs. Reproduced with permission from [44]. Copyright 2016, American Chemical Society.

Some recent representative works concerning preparation and functionalization of SiNPs are as follows. In 2013, Nishimura et al. developed a novel chemical-etching method to prepare small-sized and photostable fluorescent SiNPs for single-molecule tracking and fluorescence imaging. In this case, the single-crystal silicon wafer was first immersed into HF–HNO_3_ solution to form the SiNPs terminated with hydroxyl groups (OH–SiNPs), which were further modified with mercaptotrimethoxysilane. Then, the functionalized SiNPs were detached from the silicon wafer by using a razor blade, followed by bioconjugation with maleimide derivatives [39]. In the same year, Atkins et al. utilized the metathesis reaction of silicon tetrachloride (SiCl_4_) with sodium silicide (Na_4_Si_4_) and Zintl salts to obtain chloride-terminated SiNPs (Cl–SiNPs) with an average size of ~10 nm. Importantly, the surface of the as-prepared SiNPs can be further passivated with octylmagnesium bromide (C_8_H_17_MgBr), 4-aminophenylmagnesium bromide (C_6_H_6_NMgBr) via a Grignard reaction, respectively, providing both a nonpolar and polar surface group. In addition, the resultant Cl–SiNPs can also be passivated with aminopropyltrimethoxysilane (APTMS) to improve the crystallinity of the synthesized SiNPs [40]. In the following year, Veinot’s group reported that photoluminescence of SiNPs with a size of ~4 nm can be effectively tuned across the entire visible spectrum through surface group modification without changing the size of SiNPs. In their work, the oxide-embedded SiNPs were first prepared via disproportionation of hydrogen silsesquioxane (HSQ) at high temperature (e.g., 1100 °C). Next, the oxide matrix of the resultant SiNPs could be removed by treatment with an acid HF solution, and the hydride-terminated SiNPs (H–SiNPs) were produced. The surface of H–SiNPs can be respectively modified with alkyl, amine, phosphine, or acetal functional groups, enabling regulation of emission of SiNPs [41]. In 2014, they further synthesized the hybrid particles combining poly (diethyl vinylphosphonate) (PDEVP) and fluorescent silicon nanocrystals in three steps. The as-resultant functionalized silicon nanocrystals displayed distinct thermoresponsive behavior, attributed to the temperature-dependent coiling of PDEVP, which led to the change of the nanohybrids’ hydrodynamic radius [42]. In the same year, they also prepared D-mannose- and L-alanine-functionalized water-soluble fluorescent silicon nanocrystals (SiNCs) and used them for MCF-7 cells imaging [43]. In order to further realize facile and large-scale synthesis of SiNPs, in 2015, Zhong et al. developed a one-pot strategy based on a photochemical approach for the preparation of water-dispersible and fluorescent SiNPs (~10 g in 40 min). They achieved a high photoluminescence quantum yield (PLQY) value of up to ~25% by employing 1, 8-naphthalimide to reduce the silicon source of (3-aminopropyl) trimethoxysilane under UV irradiation [23]. In the same year, as indicated in Figure 2A, the same group further introduced an environmentally friendly method for biomimetic preparation of fluorescent SiNPs with a narrow emission spectral width (e.g., full width at half-maximum (FWHM) of ~30 nm) in a rapid (10 min) and environmentally benign manner, by using diatoms as the green silicon precursor without extra chemical reagents [38]. The authors further employed these as-prepared water-dispersible SiNPs for long-term immunofluorescent cellular imaging via bioconjugation with goat anti-mouse IgG [23,39]. More recently, Li et al. prepared nitrogen-capped silicon nanoparticles with ultrahigh PLQY of up to 90% and narrow luminescence bandwidth (e.g., FWHM of ~40 nm) for which silicon tetrabromide (SiBr_4_), rather than SiCl_4_, is employed as precursor, as presented in Figure 2B [44].

### 2.2. Preparation of One-Dimensional Silicon Nanomaterials

**Figure 3:**
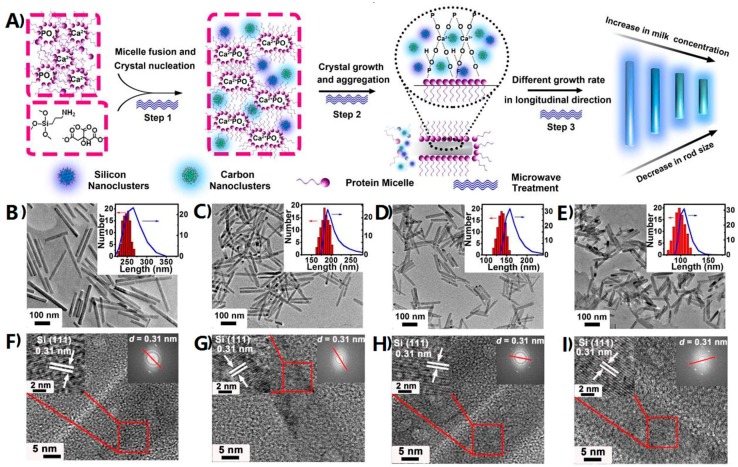
(**A**) Schematic illustration of the whole growth process of SiNRs; (**B**–**I**) TEM and HRTEM images of silicon nanorods (SiNRs) with different lengths: (**B**,**F**) 250 nm; (**C**,**G**) 180 nm; (**D**,**H**) 140 nm; and (**E**,**I**) 100 nm at different milk concentrations ranging from 1 to 4 mg/mL. Insets in (**B**–**E**) represent corresponding length distribution analysis (histograms) determined by TEM and dynamic laser light scattering (DLS) spectra (blue curves). Reproduced with permission from [47]. Copyright 2016, American Chemical Society.

Significantly different from zero-dimensional silicon nanomaterials, one-dimensional fluorescent silicon nanostructures could largely lower thresholds for optical gain and multiexciton generation owing to radiative electron-hole recombination rates and Auger recombination [45]. Consequently, these unique optical properties existing in one-dimensional nanostructures are vital for the construction of high-performance optical sensors. However, to date, there are few examples of high-quality fluorescent one-dimensional silicon nanostructures, which might be due to relatively tedious synthetic procedures, extreme experimental conditions, and low quantum yield (QY) values. For instance, Korgel et al. presented the first demonstration of one-dimensional fluorescent silicon nanorods (SiNRs) but with low QY values of ~4%–5% based on solution-liquid-solid growth and HF-etching treatment process [46]. To address this issue, Song et al. prepared size-tunable fluorescent SiNRs with the relatively high QY of ~15% and strong photostability (maintaining ~90% of the initial intensity under UV irradiation for 400 min) through one-pot microwave synthesis. As shown in Figure 3, the whole synthetic process of SiNRs contains three typical steps: micelle fusion and crystal nucleation, crystal growth and aggregation, and different growth rate in longitudinal direction. More interestingly, the as-prepared SiNRs show nearly continuous excitation wavelength-dependent emission behavior, which can be employed as a promising fluorescent label for multicolor imaging [47]. Despite these elegant works, the design and fabrication of one-dimensional fluorescent silicon nanomaterial-based sensors is still rare.

On the other aspect, numerous signal-off fluorescent sensors are designed and fabricated that take advantage of unique features of non-luminescent silicon nanowires (SiNWs), which show strong high fluorescence quenching efficiency (>90%). Nowadays, the well-established fabrication techniques of SiNWs—such as chemical vapor deposition (CVD) [48], oxide-assisted growth (OAG) [49], metal-catalyzed electroless etching [50,51], and so on—significantly facilitate the preparation of SiNW-based fluorescent sensors. In addition, SiNWs can be readily modified with metallic nanoparticles (e.g., AuNPs, AgNPs, iron oxide nanoparticles (IONPs), etc.) to produce SiNWs nanohybrids. Such processes are usually based on the galvanic displacement reactions, in which the electrons from the surfaces of SiNWs generated by HF etching (Si + 6F^−^ → SiF_6_^2−^ + 4e^−^) can effectively reduce the metal ions to form metallic nanoparticle-modified SiNWs [52,53].

## 3. Applications of Silicon Nanomaterial-Based Fluorescent Sensors

### 3.1. Zero-Dimensional Silicon Nanomaterial-Based Fluorescent Sensors

Based on the elegant work of synthesis of zero-dimensional fluorescent SiNPs, scientists have further devoted tremendous efforts to developing various kinds of fluorescent SiNP-based sensors for monitoring chemical and biological species, including heavy metal ions, explosives, glucose, pesticides, antibiotics, dopamine, intracellular pH, and so forth [54,55,56,57,58,59,60,61,62,63,64,65,66,67].

**Figure 4:**
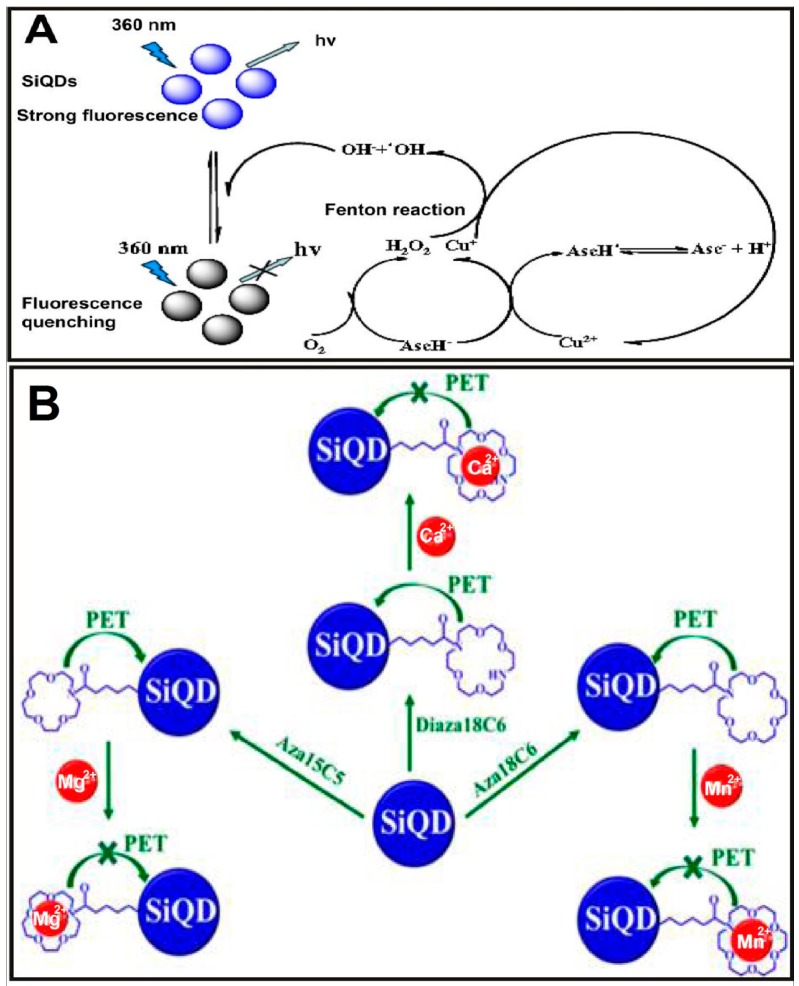
(**A**) Illustration of the strategy for the detection of Cu^2+^ based on label-free silicon quantum dots (SiQDs) and Fenton reaction. Reproduced with permission from [56]. Copyright 2014, Elsevier. (**B**) Schematic representation for the PET process and subsequent suppression of photoinduced electron transfer (PET) between the aza-crown ethers’ moiety and SiQDs in the absence and presence of metal ions. Reproduced with permission from [57]. Copyright 2016, American Chemical Society.

As for the detection of heavy metal ions, in 2014, Yu et al. developed a selective and recyclable mercuric ion (Hg^2+^) sensor based on highly fluorescent SiNPs (e.g., PLQY of ~21.6%), which was prepared through the hydrothermal treatment of APTMS in the presence of sodium citrate. Only Hg^2+^ can distinctly quench the fluorescence of SiNPs due to the strong interaction between biothiols and Hg^2+^. The SiNP-based sensor can realize rapid, label-free, and sensitive detection of Hg^2+^ with a detection range from 50 nM to 1 μM and a detection limit as low as 50 nM. More importantly, it can be regenerative for at least five cycles by the addition of ethylenediaminetetraacetic acid (EDTA) solution [55]. In the same year, Zhao et al. used silicon quantum dots (SiQDs) as novel fluorescent probes for the detection of copper ions (Cu^2+^) in a label-free, selective, and sensitive manner [56]. In their study, the SiQDs were prepared through the classical electrochemical etching method. The fluorescence of as-prepared SiQDs can be effectively quenched by hydroxyl radicals produced by the Fenton reaction between Cu^2+^ and H_2_O_2_ (Figure 4A). The fluorescence is linearly quenched as a function of Cu^2+^ concentration ranging from 25 to 600 nM. The limit of detection was as low as 8 nM, much lower than those of existing approaches [56]. More recently, in 2016, King-Chuen Lin’s group detected individual Mg^2+^, Ca^2+^, and Mn^2+^ metal ions in live cells based on turn-on fluorescent SiQDs sensors in selective and sensitive manners (e.g., the detection limit of Mg^2+^, Ca^2+^, and Mn^2+^ is 1.81, 3.15, and 0.47 μM, respectively). In their study, when SiQDs were coordinated with aza-crown ethers, the fluorescence of SiQDs was diminished, ascribed to photoinduced electron transfer (PET) from the aza-crown ethers’ moiety to the valence band of SiQDs. Remarkably, the fluorescence can be recovered in the presence of metal ions owing to the strong charge-electron binding force between the metal ions and aza-crown ether (Figure 4B) [57].

**Figure 5:**
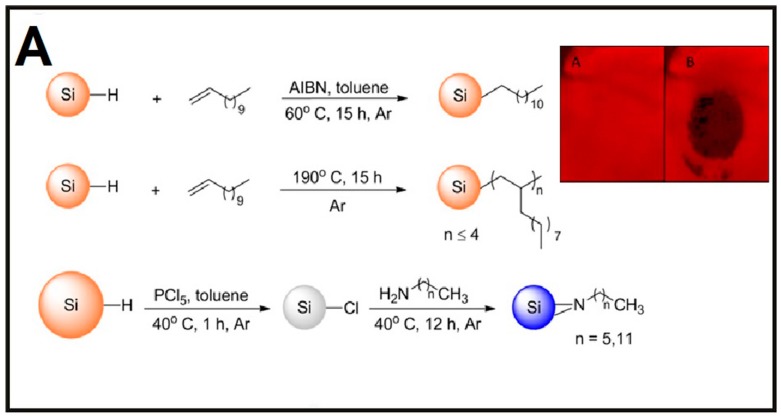

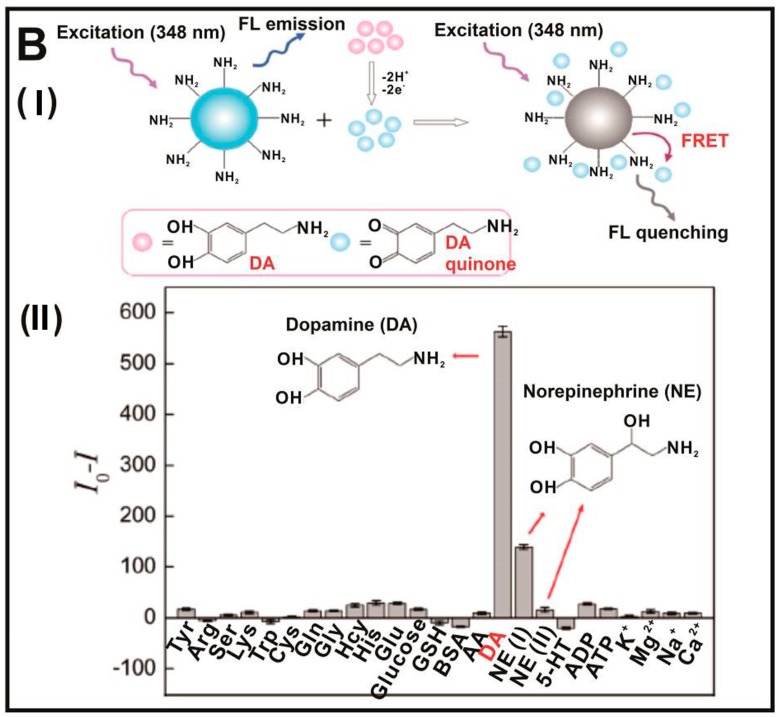
(**A**) Illustration of the strategy for the preparation of alkyl monomer—(top), alkyl oligomer—(middle), and alkyl amine—(bottom) capped SiQDs. Inset represents photographs of a quantum-dot filter paper, excited using a black light and with a 550 nm long-pass filter held in front of the camera. (A) Pristine paper; (B) the same paper showing a thumb imprint as a dark region. Reproduced with permission from ref. [61]. Copyright 2016, IOPscience. (**B**): (**I**) Schematic illustration of SiNP-based sensor for the detection of dopamine (DA); (**II**) selectivity of as-prepared SiNP-based sensor. Reproduced with permission from [62]. Copyright 2015, American Chemical Society.

As for explosives, Veinot’s group developed disposable, paper-based sensors made of dodecyl-functionalized SiNPs for the detection of explosives containing nitroaromatic, nitroamine, and nitrate ester at nanogram levels [58]. Typically, such paper-based sensors are fabricated through dip-coating filter paper in a toluene solution containing SiNPs. In the presence of mononitrotoluene (MNT), dinitrotoluene (DNT), trinitrotoluene (TNT), cyclotrimethylenetrinitramine (RDX), and pentaerythritol tetranitrate (PETN), the fluorescence of paper-based sensors was rapidly quenched. In addition, the sensors showed obvious quenching effects when exposed to solid or vapor residue of nitroaromatic compounds. The quenching results are supposed to stem from electron transfer from SiNPs to the π-orbitals of the nitro compounds. Interestingly, the quenching phenomena were reversible when exposed to volatile compounds [59]. In 2015, Ban et al. developed water-dispersible amino-capped SiNPs as fluorescent sensors for the detection of TNT in aqueous medium [59]. The authors claimed that TNT formed a complex with the surface amino groups of SiNPs through strong donor/acceptor interactions. The resultant TNT/amine complex strongly quenched the fluorescence of SiNPs via Forster resonance energy transfer (FRET) process. The authors further demonstrated that the quenching efficiencies depended on the binding affinities of nitro analytes on the SiNPs’ surface and the electron-accepting ability of the nitro compounds [59]. In 2016, Prof. Sohn’s group utilized blue-emitting SiQDs for the detection of 2,3-dimethyl-2,3-dinitrobutane (DMNB). In this case, SiQDs were obtained through the reaction of magnesium silicide with ethylenediamine dihydrochloride. The as-prepared SiQDs showed better sensitivity in the detection of DMNB compared to CdSe QDs due to their higher lying conduction band [60]. In the same year, Jonathan G. C. Veinot’s group used silicon quantum dot sensors—terminating with three kinds of surface groups (alkyl oligomer, alkyl monomer, and alkyl amine)—to detect nitroaromatics in different phases (e.g., solid, liquid, and vapor phases). In this study, the sensors were made of simple and inexpensive filter papers, dipped into silicon quantum dots solution, on which the fluorescence of silicon quantum dots is quenched in the presence of nitroaromatics due to the electron transfer quenching mechanism (Figure 5A) [61].

For other organic compounds, in 2015, Zhang et al. developed an ultrasensitive dopamine (DA) detection method with the lowest limit (~0.3 nM) assisted by water-soluble SiNPs with high quantum yield of ~23.6%, which were prepared by using a one-pot synthetic method under microwave irradiation. In particular, a quenching effect on SiNPs induced only by the DA molecule (rather than other molecules) was observed, owing to the energy transfer between SiNPs and oxidized dopamine molecules through FRET effect. Moreover, there was a linear relationship between fluorescence intensity change and DA concentrations ranging from 0.005 to 10.0 μM (Figure 5B) [62]. In 2016, Jose et al. developed a signal-off fluorescent sensor made of hexadecylamine-capped silicon nanoparticles (SiNPs) for the determination of Sudan dyes I, which were extensively adulterating food products. Sudan I can be quantified within the range of 2.91 × 10^−5^ to 4.97 × 10^−7^ M, with a detection limit of 3.90 × 10^−8^ M [63]. In 2016, Wang et al. presented dual-mode amperometric and optical-sensing strategy for the detection of glucose by using a fluorescent SiNP-coated electrode, which is more sensitive than a glucose oxidase electrode [64].

**Figure 6:**
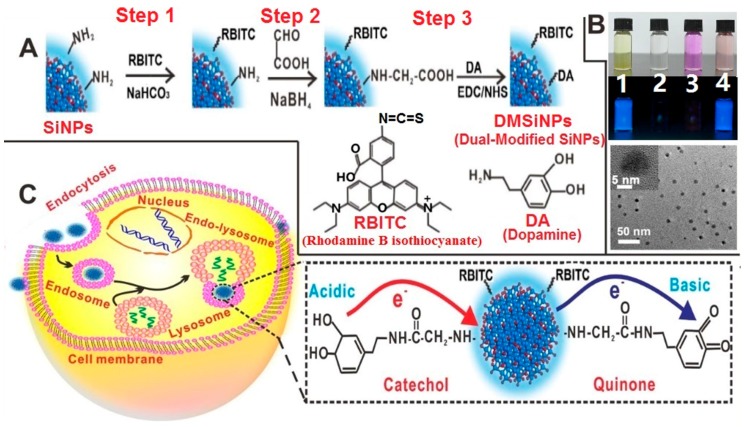
Construction of SiNP-based intracellular pH sensor. (**A**) Schematic illustration of the synthesis of sensor; (**B**) photos of SiNPs (1), dopamine (2), rhodamine B isothiocyanate (RBITC) (3), and modified SiNPs (4) in ambient environment and under UV irradiation (λ_ex_ = 360 nm) as well as TEM image of dual-modified SiNPs (DMSiNPs). Inset shows the enlarged HRTEM image of a single DMSiNP; (**C**) schematic illustration of cellular internalization of DMSiNPs. Inset in part C presents charge-transfer mechanism of DMSiNPs at acidic or basic conditions. Reproduced with permission from [67]. Copyright 2016, American Chemical Society.

Besides, for the detection of species in vitro, silicon nanomaterial-based fluorescent sensors can also be used for intracellular and in vivo analysis. For instance, in 2016, Chu et al. presented the first demonstration of fluorescent SiNP-based sensors for real-time and long-time intracellular pH (pH_i_) measurement in live cells. As revealed in Figure 6, the resultant pH sensors were made of functionalized silicon nanoparticles, which were modified with both pH-sensitive dopamine molecules and pH-insensitive rhodamine B isothiocyanate (RBITC) molecules [67]. Typically, the as-prepared sensors feature a wide-pH range response (~4–10), strong fluorescence (high PLQY of ~15%–25%), excellent photostability (~9% loss of intensity after 40 min of continual UV irradiation), and feeble cytotoxicity (cell viability of treated cells remains above 95% during 24 h treatment). Taking advantage of these outstanding features, the developed sensors are especially suitable for the ratiometric measurement of dynamic pH fluctuations and pH_i_ changes in long-term and real-time manners [67].

### 3.2. One-Dimensional Silicon Nanomaterial-Based Fluorescent Sensors

**Figure 7:**
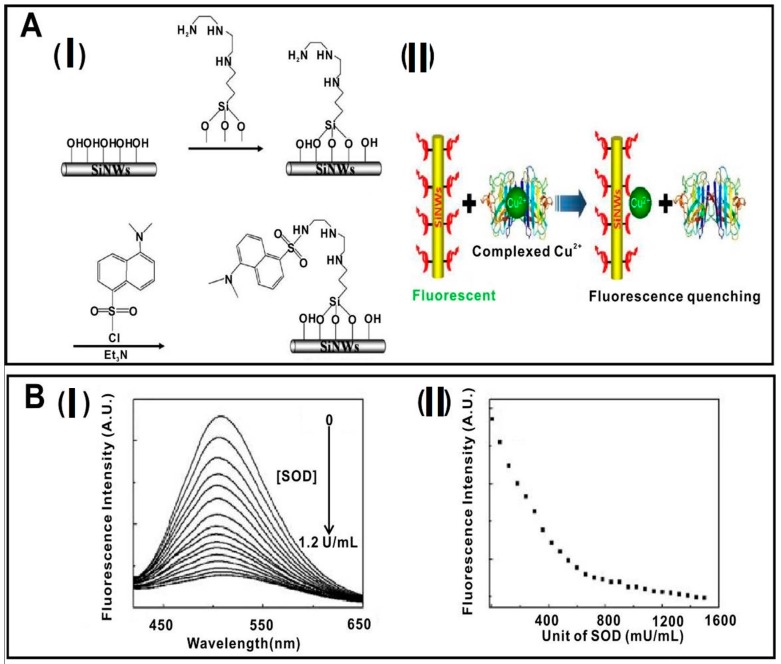
SiNW-based fluorescent sensors for the detection of complexed Cu^2+^. (**A**): (**I**) Schematic illustration of procedures of the functionalization of SiNWs; (**II**) schematic illustration of SiNW-based sensors for the detection of complexed Cu^2+^ based on fluorescence quenching; (**B**) fluorescence spectra of SiNWs-based sensors with addition of Cu-Zn-superoxide dismutase (SOD1) unit (**I**) and its corresponding titration curve (**II**); λ_ex_ = 350 nm, λ_em_ = 505 nm. Reproduced with permission from [69]. Copyright 2014, American Chemical Society.

SiNWs feature strong fluorescence quenching efficiency (>90%). Based on this advantage of SiNWs, they have been designed and fabricated as several high-performance fluorescent sensors, in which SiNWs usually acted as a quencher. For instance, as far back as 2008, Lee and co-workers constructed a kind of SiNW-based fluorescent sensor for the detection of free copper ions (Cu^2+^) in sensitive and selective manners [68]. In this case, the SiNWs were covalently modified with the fluorescence ligand of *N*-(quinoline-8-yl)-2-(3-triethoxysilyl-propylamino)-acetamide (QIOEt). Based on the observed changes of fluorescence intensities of QIOEt, low-concentration (10^−8^ M) free Cu^2+^ could be readily discriminated. Distinguishing from such SiNW-based sensors for the detection of free Cu^2+^, in 2014, Shi’s group developed a new SiNW-based fluorescent sensor, which can be used for sensing both free and complexed Cu^2+^ in liver extract, and those released from apoptotic HeLa cells [69]. As shown in Figure 7, SiNWs prepared via the conventional thermal evaporation method were covalently conjugated with 3-[2-(2-aminoethylamino)-ethylamino] propyl-trimethoxysilane (3-A) and ansyl group, in which 3-A can selectively recognize Cu^2+^ over other metal ions and numerous biomolecules. In addition, the developed sensor can effectively detect free Cu^2+^ with low detection limits as low as ~31 nM and a good dynamic range from 50 to 400 nM. More importantly, Cu-Zn-superoxide dismutase (SOD1) from real samples, as a typical copper-binding enzyme, can be quantitatively discriminated by the developed SiNW sensor, indicating this sensor featured the ability to detect the complexed Cu^2+^ [69].

**Figure 8:**
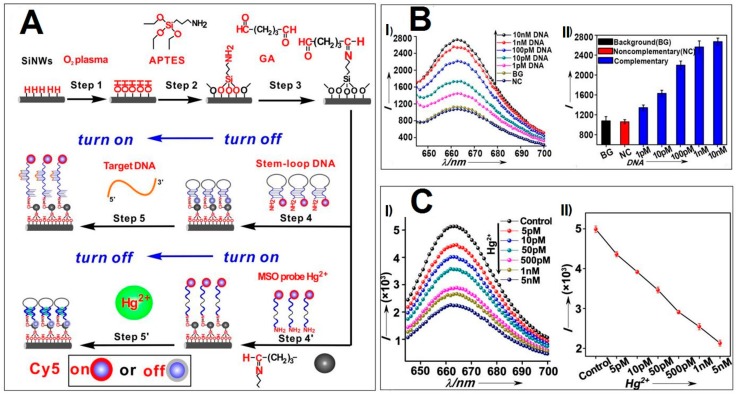
(**A**) Schematic diagram of construction of SiNW-based fluorescent sensor for DNA and Hg^2+^ detection. (**B**): (**I**) Fluorescence spectra and (**II**) corresponding PL intensities of complementary target DNA with serial concentrations. (**C**): (**I**) Fluorescence spectra and (**II**) corresponding calibration curve of SiNW-based sensors for detection of Hg^2+^ with serial concentrations. Reproduced with permission from [24]. Copyright 2014, RSC.

Besides the detection of heavy metallic ions, SiNW-based fluorescent sensors can also be workable for discriminating DNA. In 2012, Lee and coworkers utilized SiNWs as the fluorescence quencher to build a multicolor molecular beacons (MBs) sensor, which was made of AuNP-decorated SiNWs (AuNPs@SiNWs) [70]. In such a case, target DNA was readily detected via the AuNPs@SiNW-based MBs sensor by measuring the alteration of fluorescence intensity caused by the conformation change of MBs due to the DNA hybridization. Of particular significance, compared to free AuNPs with relatively poor salt stability (e.g., obvious aggregation of AuNPs would be triggered by high-concentration salt solution), the resultant AuNPs@SiNW-based fluorescent sensors featured high salt (e.g., ~0.1 M) and high temperature (e.g., ~80 °C) tolerance. In addition, the AuNPs@SiNW-based MBs could simultaneously detect multiple DNA targets at approximately picomolar levels since various DNA strands were able to assemble on the huge surface of the AuNPs@SiNWs [70]. More recently, Xie et al. further developed another type of SiNW-based fluorescent sensor based on a new assembly strategy without introduction of AuNPs [24], as shown in Figure 8. DNA strands were directly modified on the surface of the SiNWs based on the covalent conjugation with glutaraldehyde (GA), which could be further conjugated with stem-loop DNA strands. Consequently, such a sensor enables high-efficacy detection of target DNA with a limit of detection as low as ~1 pM [24].

## 4. Conclusions and Future Perspectives

In this review, we have summarized the representative works to highlight the recent success in fabrication of different dimensional silicon nanomaterial-based fluorescent sensors for various chemical and biological sensing applications. Thus far, great strides have been made in designing silicon nanomaterial-based fluorescent sensors in two different dimensions: zero-dimensional SiNP-based sensors and one-dimensional SiNW-based sensors. In particular, SiNPs with strong luminescence and robust photostability have been developed as numerous kinds of fluorescent sensors for the detection of a myriad of species. On the other hand, SiNWs/SiNW nanohybrids have also been designed and fabricated as several high-performance fluorescent sensors by utilization of high fluorescence quenching efficiency of SiNWs.

While silicon nanomaterial-based fluorescent sensors have shown great promise for myriad biological and biomedical applications, there are still many major challenges or issues in this field. First, most sensing applications are limited in laboratorial environment, thus, much effort is required to demonstrate consolidated feasibility of the silicon nanomaterial-based fluorescent sensors for practical analysis in real sample systems. Second, while various silicon nanomaterial-based fluorescent sensors are available for high-sensitivity and high-specificity bioassays, convincing and rational analytical mechanisms are still required. Third, there are few reports concerning in vitro and in vivo analysis based on silicon nanomaterials-based fluorescent sensors, severely limiting their wide-ranging applications. Finally, further improvement of the stability and reliability of silicon nanomaterial-based fluorescent sensors is still in great demand, which is of essential importance for real applications. Along with the growing understanding of the challenges, silicon nanomaterial-based fluorescent sensors would open new avenues for myriad sensing studies, and also hold high promise for widespread practical analytical applications.
